# A Study on the Developmental Features of Yi Students’ Chinese Mental Lexicon

**DOI:** 10.3389/fpsyg.2022.790215

**Published:** 2022-03-24

**Authors:** Ming Li, Lubei Zhang, Qi Zhou

**Affiliations:** School of Foreign Languages, Southwest Jiaotong University, Chengdu, China

**Keywords:** Yi students, Chinese mental lexicon, developmental features, free word association test, phonological prominence

## Abstract

Adopting free word association test, the present study investigated the developmental features of Yi students’ Chinese mental lexicon. Eighty primary school students and 85 senior high school students in two typical Yi-Han bilingual schools in Yuexi County were recruited as the research subjects. With Yi language as their L1, all the participants started learning Chinese after entering primary school. The stimuli were 108 words selected from the 9,000 most frequently used words in modern Chinese, including 36 nouns, 36 verbs, and 36 adjectives. The responses were analyzed using the framework proposed by the responses were analyzed using a three-layer framework. The results showed that (1) the mental lexicon of Yi primary and middle school students were mainly connected with each other by meanings, with form-based connection followed. Compared with previous studies, their phonological associations accounted for a relatively higher proportion, while semantic associations were relatively weakened. (2) Syntagmatic associations were of primary importance, with paradigmatic and encyclopedic associations followed. (3) While syntagmatic relation was centered on determinative and governing associations, paradigmatic relation was dominated by adjacent and reverse associations. (4) The development rate of syntagmatic associations was faster than that of paradigmatic and encyclopedic associations; no significant improvement was found on students’ adjacent, layer, homogeneous and supplement associations from primary school to high school. The findings generate important implications for Yi students’ Chinese language education.

## Introduction

Mental lexicon, as the representation of words in permanent memory, has become a focus of L2 studies in recent years. In psycholinguistics, studies on mental lexicon are not just concerned with a collection of words, but deal with how those words are stored, activated, processed and retrieved by a person when he/she wants to Al-Dala’ien et al. (2015). How is L2 mental lexicon organized in L2 learners’ minds is still a controversial issue puzzling researchers. But it is generally believed that better access to mental lexicon may better facilitate knowledge of L2 vocabularies (Zareva and Wolter, 2012; Łockiewicz and Jaskulska, 2015), and which in turn can contribute to better performance of L2 (Elman, 2004). Based on the theory of spreading activation model and word association test, numerous studies have been conducted to explore the characteristics of L2 mental lexicon constructions. While much progress has been made on researches of L2 mental lexicon, some issues need to be explored further. Some scholars assumed that the organization of L2 mental lexicon is similar to that of L1. However, other studies showed that there were great differences between the working mechanisms of L1 and L2 mental lexicon. Moreover, most of the existing studies on L2 mental lexicon have been conducted from the perspective of English as a foreign or second language. Till now few studies have looked at the mental lexicon of Chinese ethnic bilingual learners who are learning Chinese as their second language (CSL). How is their mental lexicon organized and developed as their studies in schools advance? By understanding its underlying rules and developmental patterns, teachers may become better equipped to help ethnic CSL learners’ learning Chinese vocabulary.

Yi, mainly inhabiting the south-western part of China, is a typical ethnic group that maintained their mother-tongue to a large degree. Learning Chinese as their second language, many Yi students encountered obstacles and failed to achieve a satisfactory grade. A further exploration into the dynamic features of their mental lexicon organization will provide a theoretical basis and a practical guide for Chinese vocabulary teaching in ethnic minority areas.

## Literature Review

Starting from the late 1970s, the organization of L2 mental lexicon has become a research focus in the psycholinguistic studies. Although much progress has been made, some critical issues still need to be studied further.

### Structure of L2 Mental Lexicon

As to the nature of L2 mental lexicon, there are two major assumptions. One holds that no matter L1 or L2, the mental lexicon is mainly connected semantically (Wolter, 2001). In several studies (Nissen and Henriksen, 2006; Zareva, 2007; Zareva and Wolter, 2012), no or little form-based associations have been found for both native speakers (NSs) and non-native speakers (NNSs). In Li and Jiang’s (2015) study, only 0.4% of phonological associations have been reported by native Chinese speakers and 0.5 and 2.8% by Australian intermediate and elementary Chinese L2 learners respectively. Séguin (2015, p. 92) found that “phonological similarities, being rare, would not seem to be playing an important role.” The other assumption asserts that the L2 mental lexicon is highly or even mainly dependent on form-based associations. A higher percentage of form-based associations have been found in NNSs than in NSs in many previous studies (Meara, 1978; Zhang, 2005; Fitzpatrick, 2006; Fu et al., 2009; Xie, 2009; Li and Wang, 2016b; Jiang and Zhang, 2021). After studying the NNSs in the United Kingdom, Meara (1978, p. 200) asserted that the participants were “relying heavily on the form of the word, and ignoring its meaning completely.” Several studies on Chinese English learners also reported a high percentage of phonological responses, especially in the elementary level (Xie, 2009). In Xie’s (2009) study, although the mental lexicon of advanced learners was mainly paradigmatically organized (52.4%), the phonological associations were still at a relatively high percentage (32.5%). Li and Wang (2016b) compared the word associations of Chinese English learners with those of English native speakers, and found that Chinese English learners produced 16.5% of form-related responses while English native speakers only produced 0.6% of form-related responses. Jiang and Zhang (2021) found similar trend in America. They reported that NSs of English in America produced 1.56% of form-based associations and NNSs of English with different proficiency levels, no matter high or low, both generated around 13–14% of form-related responses. These findings suggest that form-based connections play a more prominent role in the organization of L2 mental lexicon than those in L1 mental lexicon. The controversy over whether such form prominence does exist is one of the major debates in the study of L2 mental lexical organization. It seems that meaning remains a primary principle for lexical organization in both L1 and L2 (Jiang and Zhang, 2021), but form-based associations would also play an important role in L2 lexical organization.

Moreover, some other studies, mostly on EFL students in Asia, have found that encyclopedic association is one of the most important ways to organize L2 mental lexicon (Billiris, 2011; Khazaeenezhad and Alibabaee, 2013; Yu and Cai, 2014; Li and Wang, 2016b). Billiris (2011) found the percentage of encyclopedic associations produced by Korean EFL leaners accounts as high as 53%. In studies on upper-intermediate and beginners EFL students in an Iranian university (Khazaeenezhad and Alibabaee, 2013) and Chinese advanced and intermediate EFL learners (Li and Wang, 2016b), the percentages of encyclopedic associations were found to be around 20–30%. However, under the framework of “syntagmatic-paradigmatic-phonological association” or other similar frameworks (Fitzpatrick, 2007), most of the WAT studies did not single out encyclopedic associations as a category. Most of these studies focused on the comparison and contrast of paradigmatic and syntagmatic associations (Zhang, 2005; Nissen and Henriksen, 2006; Fitzpatrick, 2007; Xie, 2009; Zhang, 2010a,b; Li and Jiang, 2015; El-Dakhs, 2017; Wu, 2017; Lu and Lim, 2019). Whether is encyclopedic association an important means by which L2 mental lexicon is organized needs to be further studied.

Further, most studies investigating the organization of L2 mental lexicon either addressed meaning-based and form-based associations or paradigmatic and syntagmatic associations, only a few studies have dug deep into the subcategories of paradigmatic and syntagmatic associations (Fitzpatrick, 2007; Zhang, 2010a,b; Cremer et al., 2011; Khazaeenezhad and Alibabaee, 2013; Feng, 2014; Li and Wang, 2016b; El-Dakhs, 2017; Spätgens and Schoonen, 2020). In the few existing studies, paradigmatic and syntagmatic associations of L2 mental lexicon have been divided into different subcategories. Some studies provided a list of subcategories with a few examples, but without clear definitions. How can we better subdivide paradigmatic and syntagmatic associations? Are the classification criteria clear enough to be applicable for the subcategorization? Moreover, are the present classification frameworks dominated by English as L2 mental lexicon valid for other L2 mental lexicon? To answer these questions, further studies are needed.

### L2 Mental Lexicon and L2 Proficiency

The relationship between the L2 proficiency level and the organization of L2 mental lexicon remained unclear. Many previous studies proved that L2 learners’ paradigmatic and syntagmatic associations differed considerably from those of NSs at the lower proficiency level, however, at the higher proficiency level, a paradigmatic trend of connectivity started to dominate L2 lexical organization (Söderman, 1993; Zareva and Wolter, 2012). A shift from syntagmatic association to paradigmatic association can be observed at a higher proficiency level. Zareva (2007) found in his study that both advanced and intermediate L2 learners of English and native English speakers produced far more paradigmatic associations than syntagmatic ones. In a later study, Zareva and Wolter (2012) also found that non-native speakers of English in American universities at a higher proficiency level tended to produce more paradigmatic associations and less syntagmatic associations. This finding was supported by Xie’s (2009) study on advanced EFL learners in China and Lu and Lim’s (2019) study on Korean EFL learners. Both of the two studies found that advanced EFL learners’ mental lexicon was mainly paradigmatically organized (52.4 and 47% respectively).

However, a few other studies held that syntagmatic associations were dominant in L2 mental lexicon. Nissen and Henriksen (2006) found that Danish English L2 learners in high school produced more syntagmatic responses than paradigmatic responses. Séguin (2015) found that Croatian EFL learners at the beginning, middle, and advanced levels all had produced a large number of syntagmatic and encyclopedic associations, while the paradigmatic associations were relatively fewer; he also pointed out that the higher the learners’ L2 levels were, the more syntagmatic and paradigmatic associations and less encyclopedic associations would be given. This, in a sense, agreed with Khazaeenezhad and Alibabaee (2013), who found that although the paradigmatic shift occurred, English learners of both low and high levels produced more syntagmatic associations.

Besides, many studies have held other different views. Some studies found that the paradigmatic knowledge of L2 mental lexicon grew much faster than the syntagmatic knowledge (Zhang, 2010b,^2013^; Liu et al., 2012; Yu and Cai, 2014; Li and Wang, 2016b). Li and Wang (2016b) maintained that while advanced L2 learners had more paradigmatic associations than intermediate learners, there was no difference in syntagmatic associations. Some studies held that L2 proficiency did not affect the proportion of meaning-based and form-based responses of L2 learners (Jiang and Zhang, 2021), or the influence is not significant enough to make a quantum leap (Li and Ni, 2021). From the analysis above, it can be concluded that how L2 proficiency level affected the development of the L2 mental lexicon was still unclear.

Moreover, a range of other factors have also been found to impact the results of WAT, such as the number of stimuli, word frequency, word familiarity, word class, the concreteness of stimulus words, and the classification criteria of word associations (Zhang, 2013). It is difficult to control all these factors in a single study. Most existing studies have only considered one or two aspects (Jiang, 2002; Namei, 2004; Nissen and Henriksen, 2006; Zareva, 2007; Xie, 2009; Khazaeenezhad and Alibabaee, 2013; Li and Jiang, 2015; Séguin, 2015; Li and Wang, 2016a,b; Lu and Lim, 2019; Jiang and Zhang, 2021). A study taking an integrative consideration of more influencing factors is valuable.

### Studies on the Mental Lexicon of Chinese as Their Second Language Learners

A survey of literature showed that a large number of studies have been conducted on L2 mental lexicon. However, most of these studies were taking learners of English as subjects (Zhang, 2010a,b, 2013; Billiris, 2011; Khazaeenezhad and Alibabaee, 2013; Harrison, 2015; Séguin, 2015; Li and Wang, 2016a,b; El-Dakhs, 2017; Lu and Lim, 2019; Jiang and Zhang, 2021). The studies on CSL learners were quite few (Jiang, 2002; Li and Jiang, 2015; Wu, 2017). Jiang’s (2002) study on Chinese language learners at the University of Hawaii revealed that the higher the CSL learners’ level of Chinese proficiency was, the more paradigmatic and the fewer syntagmatic associations would be given. Further, the learners’ phonological associations had been found to be prominent. And the percentage of phonological associations decreased as learners’ proficiency level increasing. Li and Jiang’s (2015) study on Chinese learners in the University of Queensland found that Chinese learners have more associations based on the shape of Chinese characters than on the sound of Chinese characters. Their results also showed that the proportions of paradigmatic and syntagmatic associations of CSL learners at the elementary level were relatively equal. However, the mental lexicon of Chinese native speakers and CSL learners at the intermediate level were mainly paradigmatically organized. They concluded that the higher the second language proficiency was, the more similar the learners’ mental lexicon organization was to the native speakers. Wu (2017) examined the CSL learners’ mental lexicon in a Korean university and pointed out that L2 proficiency, word class and word-formation of two-character words all influenced the CSL learners’ mental lexicon organization. The percentage of syntagmatic associations was much higher in all the three proficiency level groups, and the phonological associations only accounted for a minor percentage, no more than 4%. To the authors’ knowledge, till now there was no study investigating ethnic minority students learning Chinese as their second language within China.

To sum up, considerable controversy has arisen over the organization of L2 mental lexicon and its relations to L2 proficiency. Major concerns include: (1) Whether are phonological and encyclopedic associations two major ways in constructing L2 mental lexicon? (2) What are the subcategories of paradigmatic and syntagmatic associations? (3) Among all types of word associations, which occupied a dominant position? (4) And whether is there a paradigmatic shift with the improvement of L2 proficiency? Taking Chinese Yi students as research subjects, the present study tries to explore the underlying structure of Yi students’ L2 mental lexicon as well as its developmental features. It is hoped that results of this study will contribute to the theoretical studies of mental lexicon and help educators better understand the dynamic structure of ethnic minority students’, especially Yi students’ L2 mental lexicon.

## The Present Study

Taking 165 Yi students, including 80 primary school students and 85 senior high school students, as research subjects, the present study adopts the WAT to address the following three research questions:

(1) What are the developmental features of Yi students’ meaning-based and form-based Chinese word associations?
(2) What are the developmental features of Yi students’ paradigmatic, syntagmatic, encyclopedic, orthographical, and phonological Chinese word associations?
(3) What are the developmental features of the subcategories of Yi students’ paradigmatic and syntagmatic Chinese word associations?

### The Participants

All the participants recruited for this study were Yis from Yuexi County, Liangshan Yi Autonomous Prefecture. As the seventh largest ethnic group in China, Yis maintained their mother-tongue, Yi language, to a large degree. Most of them did not learn Chinese language until they entered primary school. While Yi-Han bilingual education is the major educational model in the local society, the importance of Chinese is increasing as their studies in school progress. To explore the developmental features of Yi students’ Chinese mental lexicon, two age groups of Yi students have been selected from two typical Yi-Han bilingual schools in Yuexi. One group of students were primary school fourth-graders, and the other were senior high school students in grade 10.

After getting the consents from the school principals, the researchers entered the schools and distributed a questionnaire to gather students’ demographic information among grade 4 and grade 10 classes. Altogether, 116 and 138 valid questionnaires had been collected in grade 4 and grade 10, respectively. Excluding students who were fluent in Chinese before they entered primary school and those who were using Chinese in home, 92 students in grade 4 and 103 students in grade 10 were left. Then with a consideration of age, 5 students in grade 4 and 8 students in grade 10 were further excluded since they were more than 3 years older than the average group age. Last, 80 students in grade 4 and 85 students in grade 10 had completed the Chinese word association test and the Vocabulary Knowledge Scale. The detailed demographic information for the participants is shown in Table 1.

**TABLE 1:** Demographic information of the participants.

Group	First language	Grade	Average age	Chinese learning years	Participant numbers	Sex (male/female)
Primary school students	The Yi language	4	10.45 (*SD* = 1.01)	4.60 (*SD* = 1.70)	80	33/47
Senior high school students	The Yi language	10	17.18 (*SD* = 1.01)	10.33 (*SD* = 2.04)	85	37/48

All the Yi students in the study were native speakers of Yi language. Chinese is learned as their second language when they entered primary school. The average ages for the two groups are 10.45 and 17.18, respectively. Generally, they had learned Chinese for around 4 and 10 years. With a learning time span over 6 years, it is hoped that the dynamic feature of Yi students’ L2 mental lexicon will be captured by comparing the results of these two groups.

### Materials

#### Word Association Task

Word association task (WAT) is a widely used paradigm in psycholinguistic experiments (Colangelo et al., 2003; Vivas et al., 2019). In the common practice, participants are required to produce the first word that comes to their mind upon stimulus presentation. The responses are taken to reflect the strength of the connection between the two words or word types in memory (Nelson et al., 2000). It has been commonly employed by researchers to assess verbal learning, second-language vocabulary acquisition as well as semantic networks (Burgess, 1998; Meara, 2009; Vivas et al., 2019). However, in the current WAT research, a well-recognized challenge is the lack of a principled way of selecting stimulus words (Fitzpatrick and Izura, 2011; Yue and Fan, 2019). A general guideline followed by most researchers is stated by Meara (1983, p. 34) as “What would count as an appropriate set of stimuli depends very much on what questions you are trying to answer.” The most frequently considered criteria include word frequency, word class, word familiarity, and etc.

Combining the criteria proposed by Zhang (2010a) and Fitzpatrick and Thwaites (2020), the present study observed the following rules in selecting stimulus words:

(1) The stimuli cover words with different word frequency;
(2) The stimuli comprise words of different parts of speech, mainly nouns, verbs, and adjectives;
(3) The stimuli contain both concrete and abstract words.

All the stimulus words were selected from the 9000 most frequently used words in the online corpus of modern Chinese language built by the National Language Committee of China^1^. It is believed that the 9,000 words with the highest frequency in this corpus cover nearly all the frequently used words in modern Chinese.

**FIGURE 1:**
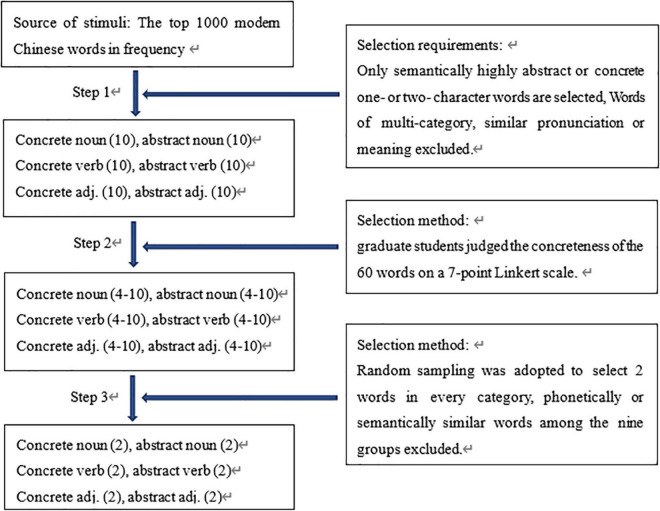
The flow chart of the selection of Group 1 stimulus words.

The selecting process consists of three steps. In the first step, 20 nouns, 20 verbs, and 20 adjectives were randomly selected from the top 1000 words, including 10 abstract and 10 concrete words in each word class. Then, the procedure was performed on words ranked 1001–2000, 2001–3000, until 8001–9000. All the words selected are one- or two-character words, of which words with multi-category, similar pronunciation or meaning were excluded. By the end, nine groups of words, each with 60 items, were gotten. In the next step, 10 graduate students majoring in Linguistics were invited to rate the concreteness of the words on a 7-point Likert scale. “1” means the word is “quite abstract,” while “7” means that the word is “very concrete”. From the score of 1–7, the concreteness increases and the abstractness decreases gradually. Before they started the rating, all the raters were required to learn and be familiar themselves with Zhang’s (2011a) definition of concrete and abstract nouns, verbs and adjectives. Examples were provided to help them understand the rules better. After all the students finished the rating, the words with an average score under 2 were labeled as abstract, and words with an average score above 6 were labeled as concrete. Others words with an average score between 2 and 6 were excluded. Thus, within each of the nine groups, 30–49 words were remaining. In the last step, four nouns, four verbs, and four adjectives were randomly selected from each group with a consideration of the concreteness of the words. That is, two concrete and two abstract words from each category (see Figure 1).

At last, 108 stimulus words were selected, including 36 nouns, 36 verbs, and 36 adjectives and there were 18 concrete and 18 abstract words of nouns, verbs and adjectives. The stimuli of nouns contained some words of superordinate category, such as “jiâ jù ‘furniture’,” “zhí wù ‘plant”’ and so on (see Appendix).

#### Vocabulary Knowledge Scale

The Vocabulary Knowledge Scale (VKS), proposed by Wesche and Paribakht (1996), was adopted to assess participants’ familiarity degree with the stimulus words. The 108 stimulus words were put into a 7-point Likert scale, with “1” meaning “never seeing or hearing the word before” and “7” meaning “knowing the word quite well.” From “1” to “7,” the familiarity degree of the word increases gradually. The results of VKS were used to ensure that participants’ familiarity with stimulus words was consistent with the word frequency. There were no high-frequency words that the participants were unfamiliar with or low-frequency words that they were quite familiar with (Zhang, 2010b).

### Procedure

Getting the principals’ consents, the researchers entered the schools and conducted a preliminary survey among grade 4 and grade 10 students. After getting the background information regarding their age, gender, and language learning experience, potential participants were recruited and grouped into 40 to 45 students a group. Each group of students was gathered in a classroom after school in the afternoon. Before the test formally began, the researchers explained the research purposes and the requirements of the tests. Examples were given to help students understand the task they were asked to do. Then the study was conducted in two steps. First, the participants were asked to complete the Chinese word association test, writing down the first word that came to their minds when seeing each stimulus word presented in writing, and Pinyin could be used when the participants had trouble in writing the Chinese character. The participants must complete the test independently within 30 min, without using dictionaries or any other reference books. Then, after finishing the Chinese word association test, the participants were asked to score the familiarity of stimulus words based on the Vocabulary Knowledge Scale.

### Analytical Framework

A survey of literature revealed that till now there were no applicable framework for analyzing the associative responses of L2 Chinese words. The only existing framework was used for analyzing Chinese as L1, proposed by Zhang and Chen (2018), which classified the associative responses into three layers based on the characteristics of Chinese (see Table 2). At the first layer, the response words are divided into meaning-based association and form-based association; at the second layer, meaning-based association is subdivided into paradigmatic, syntagmatic and encyclopedic associations, and form-based association is subdivided into orthographical and phonological associations. If the response words can co-occur with the stimulus words and form collocations, they are classified as “syntagmatic association.” If the response words and the stimuli are semantically related and belong to the same semantic field but are not necessarily mutually substitutable, they are classified as “paradigmatic association.” Responses based on the encyclopedic knowledge of personal experience, history and culture are classified as “encyclopedic association” (e.g., huǒ chái ‘match’ – xiǎo nǚ hái ‘little girl’).

**TABLE 2:** The classification framework of the associative responses of Chinese words.

The first layer	The second layer	The third layers	Examples
Meaning-based associations (Responses and stimuli are semantically related)	Paradigmatic associations	Adjacent associations	*yǒng héng-yǒng yuan* (eternal-permanent)
		Reverse associations	*guāng huá-cū cāo* (slippy-coarse)
		Layer associations	*zhí wù-xiàng rì kuí* (plant-sunflower)
		Homogeneous associations	*féi zào- xǐ yī fěn* (soap-washing powder)
	Syntagmatic associations	Indicative associations	*huǒ jiàn- shēng kōng* (rocket-blast off)
		Governing associations	*jiě pōu- shī tǐ* (dissect*-*dead body)
		Determinative associations	*píng tǎn- mǎ lù* (smooth-road)
		Sequence associations	*jǔ jué- yàn xià* (chew- swallow)
		Supplement associations	*chou- dé hěn* (ugly- extremely)
		Fixed phrases	*jiàng luò- sǎn* (land-unbrella)
	Encyclopedic associations		*huǒ chái- xiǎo nǚ hái* (match-little girl)
Form-based associations (Responses and stimuli have no semantic relation but are phonologically or orthographically similar)	Orthographical associations		*fēng gé- fēng sú* (style-custom)
	Phonological associations		*xìng qíng- xīn qíng* (disposition-mood)
Other (No response or the responses are unrelated to or just the same as the stimuli)			*chéng yīn- qiao kè lì* (cause-chocolate)

Further, at the third layer, the “paradigmatic association” is subdivided into the subcategories of “adjacent association” (e.g., cán rěn ‘cruel’ – cán kù ‘cruel’), “reverse association” (e.g., shuāi lǎo ‘old’ – nián qīng ‘young’), “layer association” (e.g., jiā jù ‘furniture’ – shā fā ‘sofa’), and “homogeneous association” (e.g., tián ‘sweet’ – là ‘spicy’; féi zào ‘soap’ – xǐ yī fěn ‘washing powder’). The “syntagmatic association” is subdivided into six categories: “indicative association,” “governing association,” “determinative association,” “sequence association,” “supplement association” and “fixed phrases.” If the responses and the stimuli can form subject-predicate phrases, they are classified as “indicative association” (e.g., huǒ jiàn ‘rocket’ – shēng kōng ‘blast off’; chéng dān ‘undertake’ – zì jǐ ‘oneself’); if they can form verb-object phrases, they are classified into “governing association” (e.g., fǔ mō ‘touch’ – tóu fa ‘hair’; jiě pōu ‘dissect’ – shī tǐ ‘dead body’); if they can form modifier-head phrases, they are identified as “determinative association” (e.g., píng tǎn ‘smooth’ – mǎ lù ‘road’; shén mì ‘mysterious’ – zǔ zhī ‘organization’); if they can form conjunction-predicate phrases consisting of two consecutive predicates, they are grouped into the category of “sequence association” (e.g., jǔ jué ‘chew’ – yàn xià ‘swallow’; xuán zhuǎn ‘spin’ – tiào yuè ‘jump’); if they can form complement phrases, they are grouped into “supplement association” (e.g., jiàng luò ‘land’ – chéng gōng ‘successfully’; chou ‘ugly’ – dé hìn ‘extremely’); if the response words can form fixed combinations or idioms with the stimulus words, they fall into the category of “fixed phrases” (jiàng luò – sǎn ‘parachute’; xuán zhuǎn – mù mǎ ‘carousel’).

Referring to the research of Li and Wang (2016b), the present study used the online corpus of BLCU Corpus Center (Beijing Language and Culture University Corpus Center, BCC) to assist the classification of response words. For example: “sī wéi – mǐn jié” can be considered as “indicative association (sī wéi mǐn jié ‘think quickly’)” as well as “determinative association (mǐn jié sī wéi ‘quick thinking’)”. The searching results of BCC showed that there were 13 records of the phrase of “mǐn jié sī wéi” and 66 of “mǐn jié de sī wéi,” which were 79 records altogether and there are 549 records of the phrase of “sī wéi mǐn jié.” Therefore, “sī wéi – mǐn jié” was classified into the category of indicative association. After classifying the response words, SPSS 23.0 was used for statistical analysis of the data.

## Results

### Developmental Features of Yi Students’ First Layer Word Association Patterns: Meaning-Based and Form-Based Associations

According to the literature, meaning-based and form-based word associations belong to the first layer. To explore the developmental features of Yi students’ first layer word associations, comparisons were made between the two groups.

Table 3 shows the results of Yi students’ word association patterns at the first layer. Participants in the two groups both produced far more meaning-based associations than the form-based and other associations. This indicates that the Chinese mental lexicon of the Yi students at primary and senior high schools is dominated by meaning-based associations and supplemented by form-based associations. The results of the chi-square test show that participants’ meaning-based associations increased significantly from primary school to senior high school, while form-based associations and other responses decreased significantly. The organization of Chinese mental lexicon develops steadily in the direction of semantic association. There was the dominance of meaning-based responses in the Chinese word association of the Yi students, however, the percentage of meaning-based associations produced by students at primary school was only 62.85%, and there were only 88.79% of meaning-based associations in the word associations of senior high school students who had learned Chinese for about 10 years. There were still 11.21% of commonly used words in modern Chinese that have not established semantic relations in Yi students’ Chinese mental lexicon.

**TABLE 3:** The results of word association at the first layer.

Association type	Group 1 (primary school) (*N* = 80)		Group 2 (Senior high school) (*N* = 85)		Chi-square test results	
			
	Frequency	Percentage (%)	Frequency	Percentage (%)	*X*^2^ value	*P*-value
Meaning-based	5430	62.85	8151	88.79	1652.46	0.000
Form-based	1876	21.71	629	6.85	813.66	0.000
Other	1334	15.44	400	4.36	622.36	0.000

### Developmental Features of Yi Students’ Second Layer Words Associations: Paradigmatic, Syntagmatic, Encyclopedic, Orthographical, and Phonological Associations

Table 4 shows the features of Yi students’ word association patterns at the second layer. The results of chi-square test indicate that participants at primary school produced more syntagmatic associations than paradigmatic (*X*^2^ = 252.09, *P* = 0.000) and encyclopedic associations (*X*^2^ = 308.63, *P* = 0.000); the percentage of syntagmatic responses of senior high school students was also significantly higher than that of paradigmatic (*X*^2^ = 1646.50, *P* = 0.000) and encyclopedic responses (*X*^2^ = 1527.53, *P* = 0.000). There were no significant differences between paradigmatic and encyclopedic associations produced by primary as well as senior high school students. The analysis above indicates that the meaning-based associations in the Chinese mental lexicon of Yi students at primary and senior high schools are dominated by syntagmatic associations and supplemented by paradigmatic and encyclopedic associations.

**TABLE 4:** The results of word association at the second layer.

Association type	Group 1 (primary school) (*N* = 80)		Group 2 (Senior high school) (*N* = 85)		Chi-square test results	
			
	Frequency	Percentage (%)	Frequency	Percentage (%)	*X*^2^ value	*P*-value
Paradigmatic	1546	17.89	1830	19.93	12.08	0.001
Syntagmatic	2424	28.06	4437	48.33	772.94	0.000
Encyclopedic	1460	16.90	1884	20.52	38.36	0.000
Orthographical	68	0.79	17	0.19	33.96	0.000
Phonological	1808	20.93	612	6.67	771.12	0.000

Moreover, as can be seen from Table 4, the proportions of syntagmatic and paradigmatic associations produced by senior high school students were higher than those produced by primary school students. Yi students at senior high school produced 20.27% more syntagmatic associations than primary school students, while the difference between the proportion of paradigmatic associations was only 2.04%. It shows that the development of paradigmatic knowledge is much slower than that of syntagmatic knowledge.

Table 4 also shows that, with the increase of grade level, the proportions of orthographical and phonological associations decreased significantly. The percentages have dropped from 0.79 to 0.19 and 20.93 to 6.67 respectively. However, the percentage of phonological associations was obviously much higher than that of orthographical associations at both stages.

### Developmental Features of Yi Students’ Third Layer Words Associations: Subcategories of Paradigmatic and Syntagmatic Associations

Table 5 shows the results of Yi students’ word associations at the third layer. In both groups, the paradigmatic relation was dominated by adjacent and reverse associations (the percentage is higher than 5.72%), supplemented by layer and homogeneous associations (the percentage is less than 3.04%); the syntagmatic relation was dominated by determinative and governing associations (the percentage is higher than 10.76%) and supplemented by indicative associations, fixed phrases, sequence and supplement associations (the percentage is less than 4.87%). The results indicate that the four meaning-based relations, namely determinative, governing, adjacent and reverse associations were much more in number or much stronger in linking strength or both in Yi students’ Chinese mental lexicon. These four meaning-based relations played leading roles in the construction of semantic networks.

**TABLE 5:** The results of word association at the third layer.

Association type		Group 1 (primary school) (*N* = 80)		Group 2 (Senior high school) (*N* = 85)		Chi-square test results	
				
		Frequency	Percentage (%)	Frequency	Percentage (%)	*X*^2^ value	*P*-value
Paradigmatic	Adjacent	613	7.09	659	7.18	0.05	0.828
	Reverse	494	5.72	707	7.70	27.87	0.000
	Layer	263	3.04	244	2.66	2.40	0.121
	Homogeneous	176	2.04	220	2.40	2.65	0.104
Syntagmatic	Indicative	200	2.31	447	4.87	83.01	0.000
	Governing	930	10.76	1653	18.01	188.38	0.000
	Determinative	1094	12.66	1924	20.96	217.78	0.000
	Sequence	5	0.06	20	0.22	8.13	0.004
	Supplement	46	0.53	49	0.53	0.00	0.990
	Fixed phrases	149	1.72	344	3.75	67.70	0.000

Table 5 also reveals that senior high school students produced significantly more reverse, indicative, governing, determinative, sequence associations and fixed phrases compared with the Yi students at primary school, with the growth rates of both determinative and governing associations exceeding 7.25%. However, the reverse, indicative, sequence associations as well as the fixed phrases increased by no more than 2.56%. The proportions of adjacent, layer, homogeneous and supplement associations in the Chinese word associations of senior high school students had no significant difference from those of primary school students. This indicates that the development of the semantic relations in the subcategories of paradigmatic and syntagmatic associations is unbalanced from primary school to senior high school. The knowledge of determinative and governing associations develops relatively faster, whereas the knowledge of reverse and indicative relations as well as fixed phrases develops slower, and the knowledge of adjacent, layer and homogenous associations remains basically stagnant. The effect of sequence and supplement associations on the structure of the lexical networks is limited, so these two associations can be ignored.

## Discussion

The results of the present study suggested the developmental path of Yi students’ Chinese word associations at three layers. The findings are of great importance for us to deepen the understanding of Yi students’ word association networks.

At the first layer, the finding that learners’ L2 proficiency level could largely be predicted by the proportion of semantic associations is consistent with many previous studies (Zhang, 2011b; Liu et al., 2012; El-Dakhs, 2017). As Liu et al. (2012, p.58) pointed out that the proportion of semantic associations could predict the developmental level of L2 lexical representation networks, which in turn could reflect learners’ L2 proficiency level, the findings of the current study also indicated that as Yi students’ Chinese proficiency improved from primary school to senior high school, the organization of their Chinese mental lexicon developed steadily in the direction of semantic association.

Moreover, compared with other Chinese L2 learners in previous studies, the percentages of semantic associations by two groups of Yi students are both at a lower level. Li and Jiang’s (2015) and Wu’s (2017) studies on Australian and Korean college students showed that after 1–2 years of Chinese learning, semantic relations could account for 88.5 and 92% of the Chinese word associations respectively. Li and Wang’s (2016a) and Zhang and Chen’s (2018) studies found that the proportions of meaning-based associations of native Chinese speakers could reach as high as 98 and 99.03% respectively. Although the stimulus words in different studies were varied in difficult level, Yi students’ Chinese learning time was much longer than those in previous studies. The minor discrepancies caused by different word selections could be neglected. The lower level of Yi students’ semantic associations indicated that there was still much room for improvement. In the Yi-Han bilingual schools, where Chinese has been used as the language for instruction, there is a reasonable prospect that the networks of Yi students’ Chinese mental lexicon can be better constructed than those of oversea Chinese L2 learners. With an effort to promote semantic relations of Yi students’ mental lexicon, it is expected that Yi students’ Chinese language proficiency can be uplifted markedly.

At the second layer, the picture is more complicated. In the present study, syntagmatic associations were found to be in a dominant position, which is consistent with the conclusions of some previous studies (Li and Wang, 2016a; Wu, 2017; Zhang and Chen, 2018). However, some other studies (Jiang, 2002; Li and Jiang, 2015) also found that Chinese word associations, either as L2 or L1, were mainly paradigmatically related. The reasons may be due to different selection of stimulus words and different subject populations. According to Nissen and Henriksen (2006) and Zhang (2006), nouns can elicit more paradigmatic responses than verbs and adjectives in L2 word associations. Jiang’s (2002) study, selecting 71 nouns and 29 adjectives as the stimulus words, may risk of overemphasizing the dominance of paradigmatic associations by using too many nouns. Further, Li and Jiang’s (2015) study only recruited 25 subjects in each experimental group, using 15 stimulus words. The number of participants and stimulus words were much smaller compared with the present study. It is reasonable to believe that with a more careful consideration of word frequency, word class and concreteness of the stimulus words, the present study can better reflect the characteristics of the organization of Chinese L2 mental lexicon, especially for Chinese ethnic minority students. Last, previous studies such as Jiang (2002), Li and Jiang (2015), and Wu (2017) did not take the encyclopedic association into consideration, which may constitute as another reason for the inconsistency with the present study.

In the present study, the proportions of paradigmatic associations of the two groups were both less than 20%, which were much lower than that of Chinese native speakers (34%) in the study of Zhang and Chen (2018). Moreover, the development of Yi students’ paradigmatic associations was also very slow, with the difference between the two groups only around 2%. This indicated that Yi students’ paradigmatic knowledge was not enhanced effectively. According to Yu and Cai (2014), the paradigmatic knowledge is related to participants’ vocabulary size, while the syntagmatic knowledge represented the participants’ the knowledge about syntax. Word pairs constituting paradigmatic associations have much subtler semantic distinctions. Thus, a higher cognitive load is required to extract response words that have paradigmatic associations with the stimulus words (Zhang and Chen, 2018, p. 81). Since participants in the present study were young, their cognitive abilities need to be developed further. Moreover, their learning environment was relatively more complex than those of other studies. Therefore, it is reasonable that paradigmatic association of Yi students in the present study was low and underdeveloped.

Further, in accordance with the results of some previous studies (Billiris, 2011; Khazaeenezhad and Alibabaee, 2013; Yu and Cai, 2014; Li and Wang, 2016b), the proportions of encyclopedic associations produced by both groups were found to be over 16%. It implied that encyclopedic association may be one of the important ways of linking L2 mental lexicon among Yi students. A positive correlation between L2 proficiency and the proportion of encyclopedic associations was also found. Although this finding contradicted some research published earlier (Séguin, 2015; Li and Wang, 2016b), it was in agreement with Khazaeenezhad and Alibabaee’s (2013) study. To get it clear on how L2 proficiency affects the frequency and the proportion of encyclopedic responses in word associations, further research is needed.

As regard to orthographical and phonological associations, phonological prominence as found in many pervious research (Fitzpatrick, 2006; Li and Wang, 2016b; Zhang and Chen, 2018; Jiang and Zhang, 2021) has been confirmed in this study as well. While the orthographical associations of both groups were less than 1%, the phonological associations produced by primary school and senior high school students were as high as 20.93 and 6.67% respectively. It was evident that with the increase of grade level, the proportion of phonological associations decreased significantly, which is consistent with the results of most previous studies. Although the proportions of phonological associations in different studies varied a lot, it may be explained by different criteria in selecting stimulus words, such as the frequency of stimulus words, the familiarity of stimulus words to subjects, and the concreteness of the stimulus words, etc.

At the third layer, among the subcategories of syntagmatic and paradigmatic word associations, four types of meaning-based relations, namely, determinative, governing, adjacent and reverse associations, were found to play leading roles in the construction of Yi students’ semantic networks. This is similar to the Chinese native speakers in Zhang and Chen’s (2018) study. However, the proportions of homogeneous and indicative associations in this study were less than 4.87%, which were significantly lower than those of native speakers in Zhang and Chen’s (2018) study (13.50 and 9.39%, respectively). This, on the one hand, may be due to the different criteria for stimulus selection. As the factor of concreteness was strictly controlled in the present study, 50% of the stimuli were abstract words and nouns of superordinate category were also included, which are relatively more difficult to trigger homogeneous or indicative associations. On the other hand, the lower percentages of homogeneous and indicative associations could also be attributed to Yi students’ lower ability to grasp the accurate meaning of the abstract words and produce subject-predicate phrases. Besides, stimulus words of lower frequency may reduce the percentage of all the types of meaning-based associations. All these factors may have some negative influence on triggering homogeneous and indicative associations.

In addition, the results also revealed that the proportions of sequence and supplement associations were quite low, not higher than 0.53%. This is in agreement with Zhang and Chen’s (2018) study, which also found that sequence and supplement associations accounted for a very low proportion in the word associations of Chinese native speakers. Thus, it is safe to conclude that the sequence and supplement associations are not the main way of semantic linking for Chinese words. Thus, in order to reduce the complexity of the hierarchical classification framework for Chinese L2 word associations, syntagmatic associations may be reduced to the following four subcategories: indicative, governing, determinative associations and fixed phrases.

## Conclusion

This study adopted the WAT to investigate the developmental features of Yi students’ Chinese mental lexicon. The main conclusions are as follows: (1) Yi students’ Chinese mental lexicon is dominated by the meaning-based associations, but the proportion of meaning-based associations can be further promoted. Syntagmatic associations seize a predominate role in the meaning-based relations, with paradigmatic and encyclopedic associations as supplements. Further, the paradigmatic associations are dominated by adjacent and reverse relations, and supplemented by layer and homogeneous relations. As for the subcategories of syntagmatic associations, determinative and governing relations take up the major roles, with indicative relation and fixed phrases followed. The proportions of supplement and sequence relations are quite low. As to the form-based associations, phonological associations accounted for a relatively higher proportion, hence the existence of phonological prominence in Chinese L2 lexical organization is confirmed. Meanwhile the proportion of orthographical associations is quite low. (2) With the increase of grade level, the paradigmatic, syntagmatic and encyclopedic associations produced by the participants increase significantly, while the orthographical and phonological associations decrease significantly. The development of syntagmatic relations is faster than that of paradigmatic and encyclopedic ones and the development of the semantic relations in the subcategories of paradigmatic and syntagmatic associations is unbalanced. The knowledge of determinative and governing associations develops relatively faster, while the knowledge of reverse, indicative relations and fixed phrases develops slower; the knowledge of adjacent, layer and homogenous associations remains stagnant.

Theoretically, the findings of this study confirmed the dominant role of syntagmatic associations in Chinese L2 word association networks. It further pointed out that phonological associations and encyclopedic associations are two major ways to organize Chinese L2 mental lexicon. Besides, it also verified the importance of context in the development of mental lexicon structure. The so-called paradigmatic shift phenomenon may not exist in certain context, for example, in the present study. Languages, learning environments and language proficiency may all be the chief factors regulating the developmental structure of L2 mental lexicon.

In practice, the findings of this study have some important implications for teaching Yi students Chinese. First of all, the mastery of commonly used Chinese words should be taken as the primary goal of Chinese education in primary and secondary schools. It is necessary to step up vocabulary teaching and broaden students’ vocabulary knowledge so as to help students build a better developed Chinese vocabulary network. Secondly, as the phonological association being found as an important way to construct the Chinese vocabulary network, teachers can cultivate students’ awareness of phonological similarity by discriminating homonyms and help students build a primary vocabulary network through phonological associations. Later, as semantic relations gradually replace phonological relations with the improvement of students’ language proficiency, efforts should be made to help students build a more complex and stable semantic network. Thirdly, certain measures can be taken to develop students’ knowledge of reverse relation, indicative relation, and fixed phrases. For example, teachers can give more examples of antonyms, subject-predicate phrases, fixed phrases and idioms using the target words. Besides, teachers can help students to develop their knowledge of adjacent, layer and homogeneous relations more quickly by listing the synonyms, hypernyms and hyponyms of the target words or words that are in the same semantic field and in the same category level with the target words. Fourthly, to expand students’ encyclopedic knowledge of vocabulary, teachers can impart more historical and cultural knowledge closely related to the target words, such as etymology, historical allusions, cultural customs, etc. Fifthly, the activities of word association can be organized regularly to check out which words are not semantically connected in students’ minds so as to make the teaching method more targeted and effective. Lastly, to cultivate their awareness of word semantic relations, activities such as vocabulary brainstorming, semantic maps painting, word list comparing and relevant words summarizing can be held.

However, it is worth noting that there are some limitations for the present study. Due to the limited space, the subcategories of encyclopedic associations are not discussed; and although the number and concreteness of noun, verb, and adjective stimuli were balanced in the research design, different association results of the three types of stimuli are not discussed separately. In the future, more studies can be conducted to explore the subcategories of encyclopedic associations and the influence of the part of speech of stimuli on lexical associations, especially the influence of the part of speech of stimuli on the subcategories of paradigmatic and syntagmatic associations.

## Data Availability Statement

The raw data supporting the conclusions of this article will be made available by the authors, without undue reservation.

## Ethics Statement

Ethical review and approval was not required for the study on human participants in accordance with the local legislation and institutional requirements. Written informed consent to participate in this study was provided by the participants’ legal guardian/next of kin.

## Author Contributions

ML, LZ, and QZ contributed to conception and design of the study, and wrote sections of the manuscript. LZ organized the database. QZ performed the statistical analysis. ML wrote the first draft of the manuscript. All the authors contributed to manuscript revision, read, and approved the submitted version.

## Conflict of Interest

The authors declare that the research was conducted in the absence of any commercial or financial relationships that could be construed as a potential conflict of interest.

## Publisher’s Note

All claims expressed in this article are solely those of the authors and do not necessarily represent those of their affiliated organizations, or those of the publisher, the editors and the reviewers. Any product that may be evaluated in this article, or claim that may be made by its manufacturer, is not guaranteed or endorsed by the publisher.

## Footnotes

1
http://corpus.zhonghuayuwen.org/

## Appendix

**Appendix 1:** The stimulus words in group 1.

Stimuli	Word class	Average score of concreteness	*SD*	Frequency	Ranking
					
					1–1000
*Sī wéi* (mind)	Noun	1.30 (abstract)	0.48	2275	557
*gài niàn* (concept)	Noun	1.60 (abstract)	0.52	1921	693
*Dòng wù* (animal)	Noun	6.70 (concrete)	0.48	2569	494
*zhí wù* (plant)	Noun	6.80 (concrete)	0.42	2535	501
*jù yǒu* (have)	Verb	1.20 (abstract)	0.42	5870	165
*yǐn qǐ* (lead to)	Verb	1.40 (abstract)	0.52	3308	345
*jìn rù* (enter into)	Verb	6.60 (concrete)	0.52	2259	560
*tīng dào* (hear)	Verb	6.60 (concrete)	0.52	1368	978
*zhǔ yào* (principal)	Adjective	1.10 (abstract)	0.32	7879	107
*tè shū* (special)	Adjective	1.20 (abstract)	0.42	1881	709
*dà* (big)	Adjective	6.70 (concrete)	0.48	20050	38
*hóng* (red)	Adjective	7.00 (concrete)	0.00	1887	705

**Appendix 2:** The stimulus words in group 2.

Stimuli	Word class	Average score of concreteness	SD	Frequency	Ranking
					
					1000-2000
*zhēn lǐ* (truth)	Noun	1.30 (abstract)	0.48	922	1447
*fēng gé* (style)	Noun	1.20 (abstract)	0.42	926	1441
*zǒng lǐ* (prime minister)	Noun	6.40 (concrete)	0.52	881	1510
*fáng zi* (house)	Noun	6.70 (concrete)	0.48	869	1529
*wéi chí* (maintain)	Verb	1.60 (abstract)	0.52	884	1503
*zhì yuē* (restrict)	Verb	1.20 (abstract)	0.42	711	1876
*biǎo yǎn* (perform)	Verb	6.70 (concrete)	0.48	1094	1232
*rán shāo* (burn)	Verb	6.50 (concrete)	0.53	749	1785
*píng děng* (equal)	Adjective	1.20 (abstract)	0.42	985	1357
*chōu xiàng* (abstract)	Adjective	1.00 (abstract)	0.00	731	1831
*duǎn* (short)	Adjective	6.70 (concrete)	0.48	1295	1053
*hòu* (thick)	Adjective	6.80 (concrete)	0.42	740	1807

**Appendix 3:** The stimulus words in group 3.

Stimuli	Word class	Average score of concreteness	SD	Frequency	Ranking
					
					2000-3000
*zhì lì* (intelligence)	Noun	1.20 (abstract)	0.42	621	2135
*pin zhì* (character)	Noun	1.20 (abstract)	0.42	528	2451
*nú lì* (slave)	Noun	6.20 (concrete)	0.42	624	2127
*gù kè* (customer)	Noun	6.70 (concrete)	0.48	475	2726
*shè jí* (involve)	Verb	1.00 (abstract)	0.00	542	2394
*chéng dān* (undertake)	Verb	1.40 (abstract)	0.52	533	2420
*fā shè* (launch)	Verb	6.80 (concrete)	0.42	595	2209
*wēi xiào* (smile)	Verb	6.90 (concrete)	0.32	588	2228
*dān chún* (pure)	Adjective	1.10 (abstract)	0.32	501	2591
*shén mì* (mysterious)	Adjective	1.00 (abstract)	0.00	497	2608
*zhí* (straight)	Adjective	6.80 (concrete)	0.42	521	2486
*yuán* (round)	Adjective	6.80 (concrete)	0.42	487	2663

**Appendix 4:** The stimulus words in group 4.

Stimuli	Word class	Average score of concreteness	SD	Frequency	Ranking
					
					3000-4000
*shēn fèn* (identity)	Noun	1.30 (abstract)	0.48	336	3666
*qīng chūn* (youth)	Noun	1.20 (abstract)	0.42	317	3852
*huǒ jiàn* (rocket)	Noun	6.90 (concrete)	0.32	372	3369
*shā mò* (desert)	Noun	6.70 (concrete)	0.48	330	3726
*jiǎ shè* (assume)	Verb	1.10 (abstract)	0.32	375	3344
*tuī lǐ* (reason)	Verb	1.40 (abstract)	0.52	312	3902
*xuán zhuǎn* (spin)	Verb	6.50 (concrete)	0.53	377	3328
*qiān dìng* (sign)	Verb	6.80 (concrete)	0.42	365	3417
*hé xié* (harmonious)	Adjective	1.30 (abstract)	0.48	367	3406
*máng mù* (blind)	Adjective	1.10 (abstract)	0.32	328	3741
*pàng* (fat)	Adjective	6.70 (concrete)	0.48	358	3465
*tián* (sweet)	Adjective	6.90 (concrete)	0.32	338	3659

**Appendix 5:** The stimulus words in group 5.

Stimuli	Word class	Average score of concreteness	SD	Frequency	Ranking
					
					4000-5000
*Lǐ zhì* (rationality)	Noun	1.30 (abstract)	0.48	294	4088
*běn néng* (instinct)	Noun	1.10 (abstract)	0.32	270	4427
*yuè qì* (musical instrument)	Noun	6.80 (concrete)	0.42	292	4113
*shū jí* (book)	Noun	6.80 (concrete)	0.42	261	4549
*pǔ jí* (popularize)	Verb	1.50 (abstract)	0.53	280	4265
*hū lüè* (ignore)	Verb	1.50 (abstract)	0.53	234	4928
*zhù shè* (inject)	Verb	6.60 (concrete)	0.52	251	4724
*zhèn dòng* (shake)	Verb	6.80 (concrete)	0.42	234	4980
*cán kù* (cruel)	Adjective	1.30 (abstract)	0.48	276	4329
*zhēn guì* (precious)	Adjective	1.10 (abstract)	0.32	268	4452
*míng liàng* (bright)	Adjective	6.70 (concrete)	0.48	290	4149
*hán lěng* (cold)	Adjective	6.60 (concrete)	0.52	249	4743

**Appendix 6:** The stimulus words in group 6.

Stimuli	Word class	Average score of concreteness	SD	Frequency	Ranking
					
					5000-6000
*xìn niàn* (belief)	Noun	1.00 (abstract)	0.00	232	5017
*líng gan* (inspiration)	Noun	1.20 (abstract)	0.42	220	5260
*huǒ chái* (match)	Noun	6.90 (concrete)	0.32	219	5272
*shǒu bì* (arm)	Noun	6.90 (concrete)	0.32	201	5673
*kě wàng* (long for)	Verb	1.40 (abstract)	0.52	231	5025
*rěn shòu* (bear)	Verb	1.40 (abstract)	0.52	230	5044
*tuī kāi* (push)	Verb	6.60 (concrete)	0.52	222	5207
*pāi shè* (shoot)	Verb	6.90 (concrete)	0.32	211	5457
*mò shēng* (strange)	Adjective	1.30 (abstract)	0.48	200	5689
*guāng róng* (glorious)	Adjective	1.20 (abstract)	0.42	197	5779
*chòu* (smelly)	Adjective	6.90 (concrete)	0.32	220	5253
*cháo shī* (moist)	Adjective	6.60 (concrete)	0.52	210	5460

**Appendix 7:** The stimulus words in group 7.

Stimuli	Word class	Average score of concreteness	SD	Frequency	Ranking
					
					6000-7000
*běn shì* (ability)	Noun	1.20 (abstract)	0.42	166	6639
*xìn yòng* (credit)	Noun	1.40 (abstract)	0.52	164	6719
*diàn qì* (electric appliance)	Noun	6.50 (concrete)	0.53	172	6442
*qiáo liáng* (bridge)	Noun	6.70 (concrete)	0.48	158	6889
*zhèn xīng* (prosper)	Verb	1.40 (abstract)	0.52	177	6281
*Jì tuō* (entrust)	Verb	1.20 (abstract)	0.42	158	6892
*jiì pōu* (dissect)	Verb	6.70 (concrete)	0.48	175	6360
*jiàng luò* (land)	Verb	6.80 (concrete)	0.42	163	6738
*zōng hé* (comprehensive)	Adjective	1.40 (abstract)	0.52	177	6282
*yong héng* (eternal)	Adjective	1.10 (abstract)	0.32	159	6854
*chǒu* (ugly)	Adjective	6.30 (concrete)	0.48	175	6357
*jiān yìng* (hard)	Adjective	6.80 (concrete)	0.42	161	6804

**Appendix 8:** The stimulus words in group 8.

Stimuli	Word class	Average score of concreteness	SD	Frequency	Ranking
					
					7000-8000
*cuò zhé* (setback)	Noun	1.20 (abstract)	0.42	141	7531
*xìng qíng* (disposition)	Noun	1.40 (abstract)	0.52	134	7853
*chē liàng* (vehicle)	Noun	6.70 (concrete)	0.48	148	7229
*jiā jù* (furniture)	Noun	6.60 (concrete)	0.52	136	7743
*píng jiè* (by virtue of)	Verb	1.50 (abstract)	0.53	152	7078
*guī huà* (project)	Verb	1.70 (abstract)	0.48	136	7744
*jià shǐ* (drive)	Verb	6.50 (concrete)	0.53	132	7938
*fǔ mō* (touch)	Verb	6.70 (concrete)	0.48	131	7997
*kōng xū* (vacuous)	Adjective	1.10 (abstract)	0.32	150	7168
*zhōng chéng* (loyal)	Adjective	1.30 (abstract)	0.48	131	7991
*shuāi lǎo* (old)	Adjective	6.60 (concrete)	0.52	140	7562
*jié bái* (pure white)	Adjective	6.80 (concrete)	0.42	137	7684

**Appendix 9:** The stimulus words in group 9.

Stimuli	Word class	Average score of concreteness	SD	Frequency	Ranking
					
					8000-9000
*chéng yīn* (cause)	Noun	1.00 (abstract)	0.00	117	8741
*shì sú* (secularity)	Noun	1.10 (abstract)	0.32	114	8961
*qiū líng* (hill)	Noun	6.70 (concrete)	0.48	119	8606
*féi zào* (soap)	Noun	7.00 (concrete)	0.00	115	8852
*yí hàn* (regret)	Verb	1.10 (abstract)	0.32	125	8280
*yù liào* (predict)	Verb	1.40 (abstract)	0.52	118	8709
*xuán guà* (hang)	Verb	6.80 (concrete)	0.42	128	8140
*jǔ jué* (chew)	Verb	6.60 (concrete)	0.52	121	8505
*zhuó yuè* (distinguished)	Adjective	1.30 (abstract)	0.48	119	8589
*xū wěi* (hypocritical)	Adjective	1.30 (abstract)	0.48	114	8940
*guāng huá* (slippy)	Adjective	6.80 (concrete)	0.42	129	8102
*píng tǎn* (smooth)	Adjective	6.80 (concrete)	0.42	123	8398
